# Gas-Thermal Spraying Synthesis of β-Ga_2_O_3_ Luminescent Ceramics

**DOI:** 10.3390/ma17246078

**Published:** 2024-12-12

**Authors:** Makhach Kh. Gadzhiev, Arsen E. Muslimov, Damir I. Yusupov, Maksim V. Il’ichev, Yury M. Kulikov, Andrey V. Chistolinov, Ivan D. Venevtsev, Ivan S. Volchkov, Vladimir M. Kanevsky, Alexander S. Tyuftyaev

**Affiliations:** 1 Joint Institute for High Temperatures, Russian Academy of Sciences, 125412 Moscow, Russia; damir.i.yusupov@gmail.com (D.I.Y.); imvpl@mail.ru (M.V.I.); kulikov-yurii@yandex.ru (Y.M.K.); a-chi@yandex.ru (A.V.C.); astpl@mail.ru (A.S.T.); 2 Shubnikov Institute of Crystallography of Kurchatov Complex of Crystallography and Photonics, National Research Centre “Kurchatov Institute”, 119333 Moscow, Russia; volch2862@gmail.com (I.S.V.);; 3 Department of Physics, Peter the Great St. Petersburg Polytechnic University, 195251 St. Petersburg, Russia; venevtsev.ivan@gmail.com

**Keywords:** gallium oxide, gas-thermal spraying synthesis, sapphire, low-temperature plasma, microscopy, X-ray diffraction, radioluminescence, ceramic

## Abstract

This paper presents the initial results of the synthesis of β-Ga_2_O_3_ luminescent ceramics via plasma gas-thermal spraying synthesis, where low-temperature plasma of an argon and nitrogen mixture was employed. A direct current electric arc generator of high-enthalpy plasma jet with a self-aligning arc length and an expanding channel of an output electrode served as a plasma source. The feedstock material consisted of a polydisperse powder of monocrystalline β-Ga_2_O_3_ with particle sizes ranging from 5 to 50 μm. The study presents the results of both theoretical and experimental studies on the heating rate and average temperature of gallium oxide particles in a plasma jet. The results of computational modelling of the synthesis process of β-Ga_2_O_3_ via plasma gas-thermal spraying are shown. The obtained ceramic samples were characterized using scanning electron microscopy and X-ray diffraction analysis. Our results indicate that the synthesis process yielded ceramics with a layered texture. The stoichiometric composition of ceramics exhibited a shift towards gallium-rich content. X-ray diffraction data demonstrated a reduction in the lattice parameters and unit cell volume of β-Ga_2_O_3_ ceramic structure. Radioluminescence spectra of β-Ga_2_O_3_ ceramics revealed an intensive emission band with a maximum at ~360 nm and non-exponential decay. The synthesized β-Ga_2_O_3_ ceramics possess potential applications in scintillation detectors.

## 1. Introduction

Gallium oxide Ga_2_O_3_ (β-phase) is one of the most studied and prospective materials due to its band gap of 4.8 eV and high electric breakdown field strength of 8 MV/cm [1,2,3,4]. β-Ga_2_O_3_ crystallizes into a monoclinic structure, where gallium ions have both octahedral and tetrahedral coordination. Although it has several polymorphs, the β-phase of gallium oxide is considered the most stable due to the coordination polyhedra having adjacent edges. β-Ga_2_O_3_ is utilized in power electronics [5], as a detector material for UVC [6] and X-ray [7] wavelength range, and in high-temperature gas sensors [8]. Notably, devices based on gallium oxide with high operational stability are quite promising for use in environments with intense radiation exposure, such as space exploration studies. Despite the high demand, economically viable technology for the growth of β-Ga_2_O_3_ bulk crystals is not yet available. To date, the maximum size of synthesized crystals is limited to 10 cm [9]. A significant challenge in the melting technologies used for crystal synthesis is the high-rate dissociation of initial gallium oxide powder at temperatures above 1200 °C [10]. Gallium oxide dissociates through the following sequence of reactions:

Ga_2_O_3_(s) → 2GaO(g) + ½O_2_(g);
2GaO(g) → Ga_2_O(g) + ½O_2_(g);
Ga_2_O(g) → 2Ga(g) + ½O_2_(g). (1)

Active gallium atoms interact with the crucible material, resulting in crucible degradation and burning, while contaminating the growing crystal with crucible material. Thin film materials based on gallium oxide are considered as an alternative for bulk crystals. Although thin film technology partially addresses the problem, it presents several challenges, such as uniformity and thickness control, adhesion and delamination issues, equipment cost, scalability of the synthesis process, surface smoothness, control of synthesis process and reproducibility of properties. Ceramic technologies are continuously developing, and ceramics today are firmly establishing their niche in materials science. Despite this, there are relatively few studies on β-Ga_2_O_3_ ceramics [11,12], and the efficiency of the methods used in these works remains relatively low. Overall, all known synthesis methods are labor-intensive, making the search for more accessible technologies particularly important. In this study, we present the first attempt to adapt the plasma gas-thermal spraying method for the synthesis of ceramic materials. Plasma gas-thermal coatings have long been used to protect surfaces from corrosion [13]. Essentially, the method involves heating and transporting particles of sprayed material via plasma flux to create a protective layer on the substrate surface. As other studies have shown [14], the characteristic structure of plasma gas-thermal coatings has a laminated character and consists of disk-shaped crystallites. The percentage of closed porosity ranges from 2% to 12% depending on the spraying method, comparable to that of ceramics obtained by traditional methods. A key difference between traditional methods of plasma gas-thermal spraying of coatings and the synthesis of isolated β-Ga_2_O_3_ ceramics is the need to minimize adhesion of the coating to the substrate and facilitate its delamination during cooling. This can be achieved by selecting appropriate materials and varying the surface topography of the substrate.

Plasma treatment is highly efficient, economically viable and environmentally friendly. The main types of plasma are high-temperature plasma, with temperatures exceeding 10^6^–10^7^ K, and low-temperature plasma, with temperatures ranging from 10 to 10^4^ K [15]. Low-temperature plasma can be further divided into thermal and non-thermal (cold) plasma. High-temperature plasma, due to its powerful energy flux, can have a destructive effect on materials. In contrast, cold plasma is characterized by low ion and inert gas temperatures of 300–400 K, although the electron gas temperature can be quite high and can reach up to 10^5^ K. Cold plasma is used for targeted modification of material surface properties [16,17]. An intermediate type of plasma is low-temperature thermal quasi-equilibrium plasma, with temperatures around 10^4^ K [18,19]. This type of plasma can be used as a universal thermal carrier with the possibility to control the degree of influence on materials. In [20], the results of mathematical modelling of particle heating by plasma flux during their motion from the plasma torch nozzle section to the substrate surface are shown. The particles were heated by the combination of convective and thermal radiation heat transfer mechanisms. The calculations were carried out considering that the exposed particles were approximately spherical in shape and that the heat flux exposing the surface of the sphere was uniform. As a result, it was shown that the particles larger than 60 μm melted and reached a temperature of 2000 °C during their transit through the plasma with a temperature of 10^4^ K. According to [21], for aluminum oxide, which chemical and physical properties are similar to those of gallium oxide, the lower boundary of the plasma temperature required to melt the particles is in the range of 4000–5000 K, depending on particle size.

To increase the safety of the plasma treatment, argon or nitrogen is typically used as the plasma-forming gas. Nitrogen exhibits relatively high thermal conductivity, a high breakdown voltage, and, consequently, significant thermal efficiency across various plasma torch operating modes. Therefore, the mixture of argon and nitrogen is considered the most appropriate choice for the gas-thermal spraying synthesis of ceramics. The addition of nitrogen results in an increase in arc length, thereby facilitating interaction with the plasma zone.

The melting stage is crucial for ensuring the uniformity and reducing the porosity of ceramics. To form a continuous, layered ceramic structure, it is essential to sequentially deposit accelerated and fused particles that undergo plastic deformation upon impact with the substrate. The temperature of 2000 °C exceeds calculated temperature of 1200 °C, at which the dissociation of gallium oxide begins. However, it is important to note that during the modelling of the particle heating process in the plasma jet, key factors such as the maximum temperature, the average length of the discharge gap, and monodispersity of the particles were considered. In practice, variations in the technological parameters of the treatment process are possible.

Among the most critical properties of synthesized ceramics are spectral and decay kinetics characteristics. The high density, thermal and chemical stability, strong theoretical breakdown electric field, radiation tolerance, and numerous other notable properties make β-Ga_2_O_3_ a highly desirable material for X-ray detection applications [7].

This paper presents the initial results of the synthesis of β-Ga_2_O_3_ luminescent ceramics via plasma gas-thermal spraying synthesis, where low-temperature plasma of an argon and nitrogen mixture was employed. A direct current electric arc generator of high-enthalpy plasma jet with a self-aligning arc length and an expanding channel of an output electrode served as a plasma source. The obtained samples of β-Ga_2_O_3_ ceramics were characterized using a combination of microscopic, spectroscopic and structural-phase analysis techniques.

## 2. Materials and Methods

**Figure 1:**
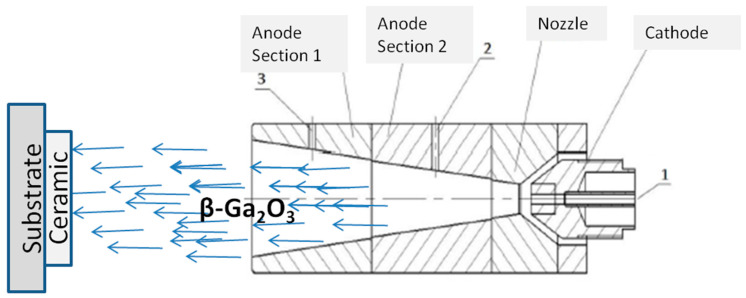
Schematic illustration of the gas discharge path of the plasma torch designed for spraying: 1—powder feed to cathode area, 2—into the anode region, 3—below the zone of the binding of arc to anode.

A commercially sourced, polydisperse gallium oxide powder (99.99%, OChW, Moscow, Russia) served as a feedstock material, with individual particles reaching a maximum size of approximately 50 μm. The powder for the spraying was delivered along with the transporting gas to the anode (Figure 1) of direct current low-temperature plasma generator (mass flow rate of plasma-forming gas 1.5 g/s, mass flow rate of transporting gas 0.2 g/s, arc current 250 A), developed on the basis of a plasma torch with self-aligned arc length, vortex stabilization, and an expanding channel of the output electrode [15,16]. The plasma-forming gas consisted of a mixture of 0.7 Ar and 0.3 N_2_ by mass. Sapphire crystals were used as a substrate material. To determine the velocity and temperature of the particles, a measuring system based on IR matrix, proposed in [22], was utilized. High-speed video cameras were used to capture a series of images to measure the particle velocity and temperature according to the following principle; at a given camera exposure time, maximum velocity can be determined: v_max_ = h/τ (h—the height of the frame, τ—the exposure time). The minimum temperature that can be determined using this system is dependent on the camera exposure time, with longer exposure times allowing for the measurement of lower temperatures. Prior to the experiments, the measuring system was calibrated using a reference blackbody over a temperature range of 800–3000 K.

The open porosity of samples was determined using a hydrostatic weighing technique. Microscopic studies were conducted using a JCM-6000 (JEOL, Tokyo, Japan) desktop scanning electron microscope, equipped with an energy dispersive X-ray (EDX) microanalyzer. X-ray diffraction (XRD) analysis was carried out on an X’PERTPRO diffractometer (PANalytical, Almelo, The Netherlands) in the Bragg–Brentano reflection geometry, utilizing CuK_α_ radiation (λ = 1.54 Å) with a Ni β-filter. Radioluminescence spectra (RL) were measured in a reflection geometry under continuous excitation from a tungsten anode X-ray tube (40 kV, 10 mA). The spectra were recorded using MDR-2 monochromator coupled with a Hamamatsu H8259-01 photon counting head. The obtained spectra were compared with those of the YAG:Ce crystals previously studied in [23]. The RL decay kinetics were measured under pulsed X-ray excitation using the TCSPC method with the setup described in [24]. The excitation pulse width was approximately 0.8 ns.

## 3. Results and Discussion

### 3.1. Theoretical Calculations of Velocity and Heating Temperature of Particles in Plasma

To estimate the degree of particle heating, we assume that primary heating occurs within the core of the plasma jet. In this region, characterized by a length b, we consider the plasma temperature T_g_ and velocity V_g_ of plasma to be constant. For a spherical particle with a diameter of D_p_, the equations for the velocity change along the jet axis V_p_ and its enthalpy H_p_ can be expressed as [25]:

$$\frac{dV_{p}}{dt}=\frac{V_{g}-V_{p}}{\tau_{D}},$$

(2) $$\tau_{D}=\frac{1}{18}\frac{D_{p}^{2}\rho_{p}}{\mu_{g}\phi_{s}},$$

$$\frac{dH_{p}}{dt}=\alpha \pi D_{p}^{2}\left(T_{g}-T_{p}\right),$$

$$\alpha =\frac{K_{g}}{D_{p}}Nu,$$

where $\tau_{D}$—the particle acceleration time constant, which is approximately 10^−2^ s during plasma treatment; K_g_, μ_g_—heat conduction and viscosity of plasma, respectively; ρ_p_—density of particle material; α—heat dissipation coefficient; Nu и $\phi_{s}$—Nusselt number and the correction to the Stoke’s drag coefficient, respectively.

Assuming the initial velocity of particles in the flux of plasma-forming gas is negligible, the relationship between the particle velocity in plasma V_p_ and the plasma velocity V_g_ can be expressed as:

(3) $$V_{p}=V_{g}\;(1-e^{\frac{-t}{\tau_{D}}}),$$

For small t << $\tau_{D}$, the time Δt required for a particle to traverse a distance b in the plasma can be obtained by expanding of the exponential function (3) into a Taylor series:

(4) $$\Delta t=\sqrt{\frac{2b\tau_{D}}{V_{g}}},$$

The process of particle heating is typically more rapid than their acceleration in the plasma flow. According to (3), the stabilization of particle velocity in the plasma flow is achieved within a time frame of <<1 s. The dominant processes occurring in the plasma are related to the heating of the particles.

The heat amount transferred to the particle during time Δ*t*:

(5) $$\Delta H=\alpha \pi D_{p}^{2}(T_{g}-T_{p})\Delta t,$$

From (2), (4) and (5), considering T_p_ << T_g_, it follows that:

(6) $$\Delta H=k_{g}T_{g}\sqrt{\frac{b}{V_{g}\mu_{g}}}\frac{Nu}{\sqrt{\phi_{s}}}\frac{\pi }{3}D_{p}^{2}\sqrt{\rho_{p}},$$

In (6), the factor *k_g_T_g_*$\sqrt{\frac{b}{V_{g}\mu_{g}}}$ represents the total energy transferred to the particle within the plasma. The parameter Nu characterizes the average intensity of convective heat transfer between the particle’s surface and the gas flow. The parameter ϕ_S_ depends on the Reynolds number, which describes the flow of gas around the particle. The ratio $\frac{Nu}{\sqrt{\phi_{s}}}$ has a weak dependence on the plasma jet parameters and, under atmospheric pressure, varies within the range of 2.6–2.9 for both argon and nitrogen. The factor $D_{p}^{2}\sqrt{\rho_{p}}$ characterizes the particles themselves. For the temperature estimation, values κ_g_~1 Wm^−1^K^−1^ and μ_g_~10^−4^ kg m^−1^s^−1^ were used [26]. The specific heat capacity increases with temperature. Due to the lack of data on the specific heat capacity of gallium oxide at high temperatures in the literature, a three-fold increase (c_p_ = 3 × 0.49·10^3^ j kg^−1^K^−1^) for temperatures above 2000 K was considered during calculations, similar to other materials. As a result of the calculation according to (6), for Ga_2_O_3_ particles with a size of 50 μm, fed through hole 3 into the anode section 1 (Figure 1) and traversing a distance of b = 17 mm at an average plasma mass temperature of 3000 K, the heating temperature is approximately 2500 K. Particles with sizes less than 30 μm according to (6) are expected to evaporate completely. The same goes for all the particles entering the plasma through hole 2 into anode section 2, and even more so for particles traversing through the cathode zone (hole 1).

### 3.2. Experimental Study of Velocity and Temperature of Particles in Plasma

Several powder feeding methods were considered: feeding through hole 3 in the first section of the anode; through hole 2 in the second section of the anode; and through hole 1 in the cathode zone. As anticipated, feeding the powder near the cathode tip and through hole 2 resulted in the significant overheating and evaporation of the initial powder. A molten precipitate was observed on the inner walls of the anode zone. The best results were achieved by feeding the powder into hole 3 in the first section. The obtained samples had mechanical strength and low open porosity (approximately 5%). It is worth noting that the minimum achievable open porosity using the gas-plasma deposition method is araound 2%.

**Figure 2:**
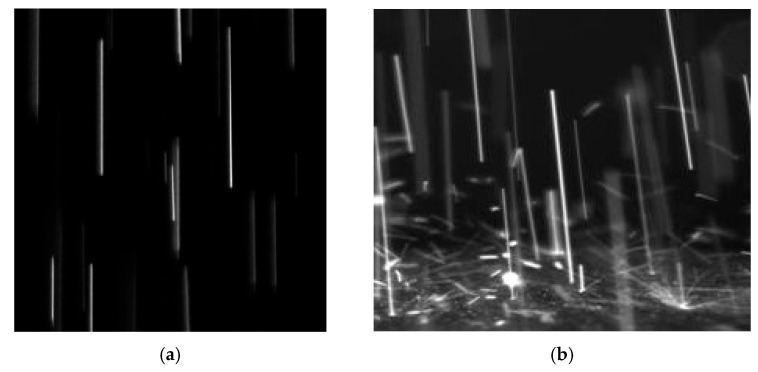
Glowing tracks of Ga_2_O_3_ particles during the motion (**a**) and before the collision with sapphire substrate (**b**).

**Figure 3:**
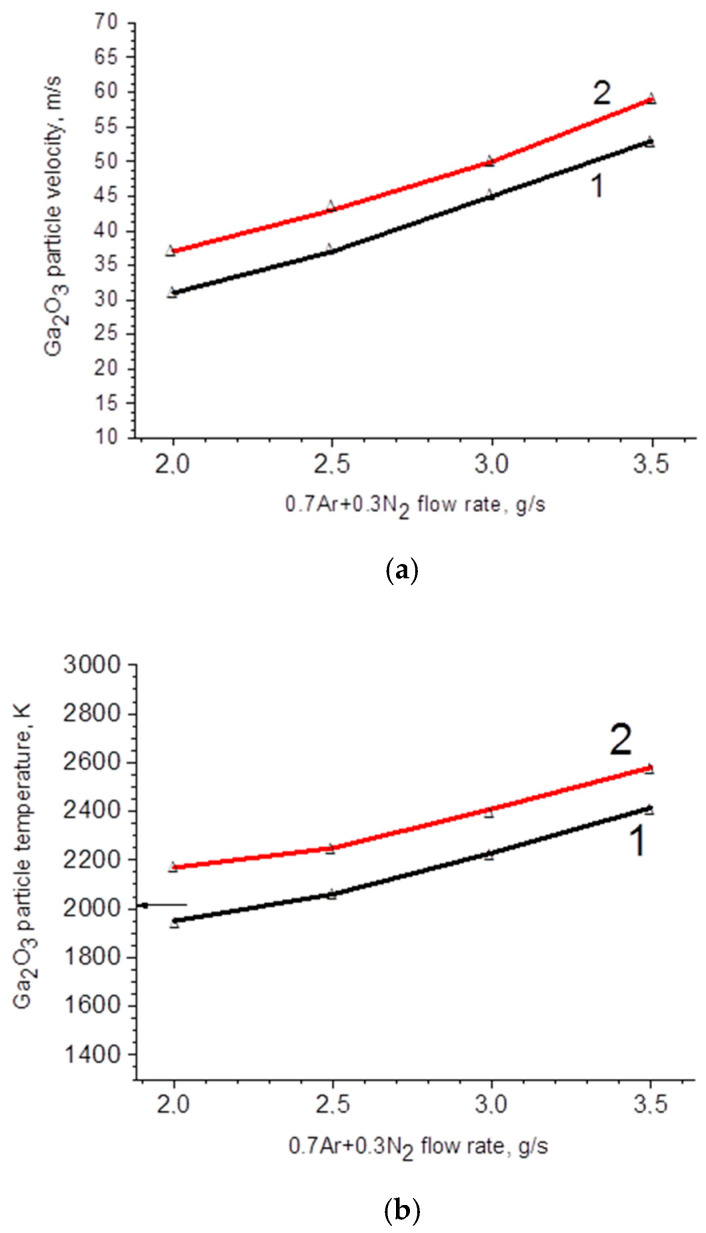
Dependence of temperature (**a**) and particle velocity (**b**) of Ga_2_O_3_ on arc current and plasma-forming gas mass flow rate. The arrow points at the melting temperature of β-Ga_2_O_3_. Designation on the graph: 1–250 A, 2–350 A.

After the data processing, the relationships between the average particle velocity, temperature of the particles and the plasma-forming gas mass flow rate and arc current were determined (Figure 2 and Figure 3).

**Figure 4:**
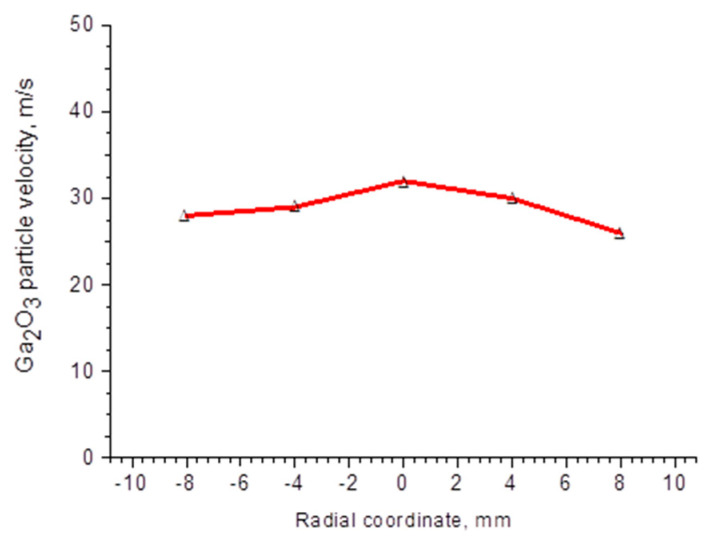
Radial distribution of particle velocity of Ga_2_O_3_. Arc current value is 250 A.

Figure 3a demonstrates an increase of particle velocity with an increase in transferring gas mass flow rate and arc current, which is consistent with (3). Similarly, an increase in particles temperature with an increase in the arc current in a discharge path can be observed (Figure 3b). This may be attributed to the increase in the total energy that gallium oxide particles acquire in the plasma, as represented by the factor *k_g_T_g_*$\sqrt{\frac{b}{V_{g}\mu_{g}}}$ in (6). Moreover, an increase in particle temperature under a direct current and an increase in the gas mass flow rate is observed. As the flow rate increases, the arc burning voltage also increases, contributing to the rise in the total energy consumed by the particles. The radial distribution (Figure 4) of particle velocity relative to the plasma axis exhibits a sufficiently high uniformity, which is crucial for the formation of a uniform ceramic structure.

The results obtained in the experiment can be compared with the theoretical calculations [23] for the aluminum oxide particles. For similar particle sizes, the average velocity of gallium oxide particles is lower than that of aluminum oxide particles. This may be due to the higher density of gallium oxide particles. The increased density results in a longer transit time for gallium oxide particles within the discharge path, and, consequently, they absorb more heat. However, given the similar thermophysical and geometrical parameters of gallium oxide and aluminum oxide particles, their heating temperatures are inversely proportional to $\sqrt{\rho_{p}}$, indicating that denser gallium oxide particles will heat up less.

According to (6), the average temperature of gallium oxide particles should be approximately 2500 K, which is higher than the temperature obtained in the experiment. However, several assumptions were made during the calculations according to (6) due to the lack of reliable data in the literature. Moreover, the losses by radiation in plasma and the heat losses on desorption from the gallium oxide particle surfaces were not considered in the model. The heat losses on desorption can be estimated according to:

(7) $$\Delta H_{\mathrm{vap}}=\frac{3q_{s}}{\rho \frac{dr}{dt}},$$

where ΔH_vap_—the heat of evaporation,$\;q_{s}$—the heat flux to the particle surface, $\frac{dr}{dt}$—a decrease in the particle radius due to evaporation. However, the values $q_{s}$ and $\frac{dr}{dt}$ are challenging to determine experimentally within the framework of our research. Nevertheless, the heat losses due to desorption could be significant, considering the intense evaporation of gallium at temperatures above 1200 °C.

According to the experimental data (Figure 3b), at the minimum gas mass flow rate of 2 g/s and minimum arc current of 250 A, the average temperature of the particles reaches the melting point. Moreover, it is noteworthy that with the decrease in the particle diameter and the increase in the relative proportion of surface atoms, the melting temperature of particles decreases significantly. Additionally, this analysis does not account for the separation of particles in plasma, where particles of different compositions follow distinct trajectories, and, consequently, have varying time of being in the high-temperature region of plasma. To achieve a more uniform microstructure, it is essential to rotate the substrate.

### 3.3. The Gas-Thermal Synthesis of β-Ga_2_O_3_ Ceramics

In the initial experiments, the following parameters were used: a gas mass flow rate of 2 g/s and an arc current of 250 A. However, the optimal quality of the β-Ga_2_O_3_ ceramics was achieved by reducing the mass flow rate of the transporting gas to 1.6 g/s.

**Figure 5:**
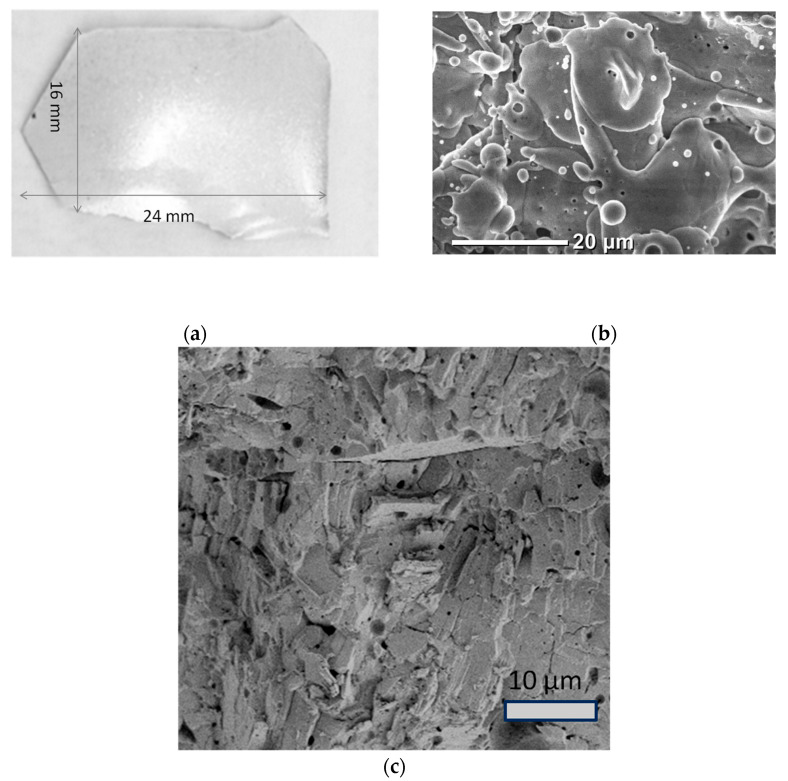
β-Ga_2_O_3_ ceramics: optical microscope image (**a**); SEM image of the surface (**b**) and cross-cut (**c**).

During the gas-thermal synthesis process, a ceramic sample of gallium oxide was formed with a size of about 2.5 cm (Figure 5a).

Scanning electron microscope (SEM) was used to investigate the topography and the cleaved facet of the sample (Figure 5b,c). By analyzing the image of the cleaved facet, an estimation of the sample’s density and uniformity can be made. Pores of various shapes with lateral sizes of up to several microns can be observed on the cleaved facet. The microstructure of synthesized gallium oxide ceramic samples can be characterized by its laminarity, which is a feature of the plasma gas-thermal spraying method. The layered structure is formed with disk-like flattened globules with sizes of up to several tens of microns, oriented along the ceramic plane. Rounded microstructures, mostly spherical and elongated in shape, with a wide range of lateral sizes (1 to 5 μm) are observed on the sample surface. Particles with minimal sizes may have formed through the collision and spreading of large, fused particles on a growing surface. As previously discussed, the separation of particles with different sizes during their motion in plasma can significantly impact the resulting microstructure. Particles with the smallest diameter experience the greatest velocity loss and temperature decrease in the gap between plasma core and the substrate. Therefore, the presence of rounded formations up to 5 μm in diameter is likely due to the transfer of small gallium oxide particles through the plasma region and their deposition on the substrate. Due to the significant deviations in trajectories, small particles of the powder do not enter the core of the plasma and melt after hitting the substrate. This assumption is supported by the formation of etching pits on the surface of ceramics, which indicates the effect of high temperature.

According to the EDX data (Table 1), the initial powder had a stoichiometric composition. However, a gallium surplus is present in the ceramic sample. Additionally, a minor Si content of less than 0.5% was observed, which could have originated from the sapphire substrate.

**Figure 6:**
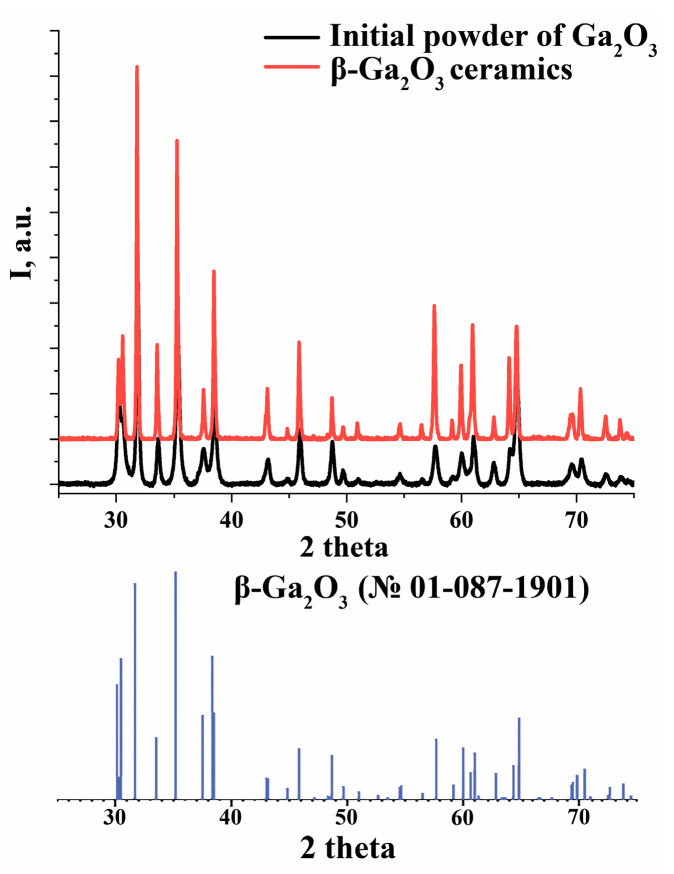
XRD patterns of the initial powder of Ga_2_O_3_ (black line) and ceramic sample of β-Ga_2_O_3_ (red line).

The XRD analysis (Figure 6) of the initial Ga_2_O_3_ powder and β-Ga_2_O_3_ ceramics indicates the presence of a single phase, β-Ga_2_O_3_ (PDF card N°01-087-1901 [27]). According to previous studies [28], all gallium oxide modifications transform into β-Ga_2_O_3_ at temperatures above 870 °C, making the formation of β-Ga_2_O_3_ during the gas-thermal plasma synthesis a typical outcome. The sizes of coherent scattering regions (CSRs) in the samples were estimated using the Debye–Scherrer equation:

(8) $$D=\frac{k\cdot \lambda }{\beta \cdot \cos\theta },$$

where *D* is the average size of CSRs, which can be less than or equal to the grain size; *k* is the dimensionless particle shape coefficient (Scherrer constant); *λ* is the wavelength of copper X-ray radiation; *β* is the full width at half maximum of the diffraction peak; *θ* is the Bragg angle.

The evaluation based on (8) showed that the average size of the CSRs in the obtained ceramics is larger than that of the initial powder. Table 2 presents the average sizes of CSRs for both the initial powder and resulting ceramics along several crystallographic directions. These results indicate a significant increase in the degree of crystalline perfection of ceramic samples compared to the initial powders of the same phase. Gallium oxide powders are typically synthesized via chemical methods that involve drying and calcination stages. As a result, the crystalline structure of the initial powder is often distorted due to the presence of defects, which cause elastic stress and lead to microdeformations and fluctuations in microstress distribution. However, during high-temperature treatment, a partial relaxation of these stresses occurs, resulting in an improvement in crystalline quality.

At the same time, the XRD results indicate a slight distortion of the crystal lattice. The calculated lattice parameters for the initial powder and gallium ceramics are presented in Table 3. The unit cell volume (V) for the ceramic sample is 0.485% smaller than the reference value, whereas the initial powder deviates by 0.985%. According to Table 1, the initial sample had a stoichiometric composition, whereas the synthesized ceramic sample was gallium-rich. The discrepancy in lattice parameters for the initial powder can be associated with the microstresses introduced into the powder during the preparation stage, including grinding and drying. In contrast, the ceramic sample’s smaller V compared to the reference value can be associated with the increased volatility of gallium during high-temperature synthesis, resulting in gallium atoms accumulating at grain boundaries. Additionally, a slight shift in the peak positions towards larger angles may indicate an excess of oxygen, consistent with Vegard’s rule and confirmed by the EDX results.

### 3.4. Modelling of Gas-Thermal Synthesis Process of β-Ga_2_O_3_ Ceramics

The gallium oxide particles heated in the plasma jet collide with the surface, spread out, adhere to topographical defects of the underlaying layer, and cool. The deformation value of a particle k_d_ can be described as:

(9) $$k_{d}=\frac{h_{p}}{D_{0}},$$

where $h_{p}$—height of the particle; $D_{0}$—initial particle diameter. According to (9) and Figure 5b, for the bulk of particles, k_d_ << 1. The coefficient of structural ordering k depends on k_d_ in accordance with [14]:

(10) $$k=\sqrt{\frac{2}{3k_{d}^{3}}},$$

As the value of к increases, the structural ordering also increases. According to (10) and Figure 5b, the value of k can be estimated for different particles, and is found to fluctuate within the range of 45–82. The magnitude of particle heating due to collision and kinetic energy transfer is ΔT ˂ 0 K, which is negligible under our conditions of thermal plasma processing.

The minimum velocity at which incoming gallium oxide particles deform upon the collision can be calculated according to [14]:

(11) $$v\;>\;2\sqrt{\frac{\alpha ({\sqrt{6k_{d}}+k}_{d}^{-1}-3)}{\rho D_{0}}},$$

where α—specific surface energy of gallium oxide ~0.5 N/m [29]. According to (11), for the particles with sizes of 15–50 μm, a velocity of ~15–30 m/s is sufficient to deform the incoming particles. The experimental data (Figure 4) confirm that the particles entering the plasma jet accelerate and reach the aforementioned velocity values.

**Figure 7:**
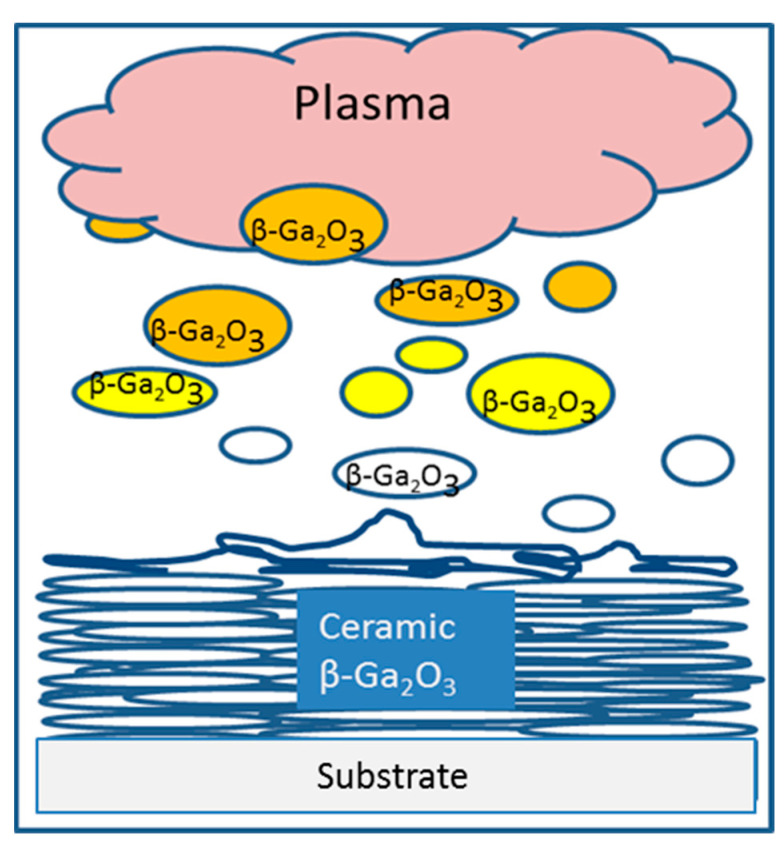
Scheme of the gas-thermal spraying synthesis of β-Ga_2_O_3_ ceramics.

The crystallization time of a particle **t_cr_** can be estimated according to:

(12) $$t_{\mathrm{cr}}=\frac{{h_{p}}^{2}}{4z\beta^{2}},$$

where β—the square root of the characteristic equation, z—thermal conductivity coefficient of particle material. For many materials, β~0.5, and the thermal conductivity coefficient of oxides is around 10^−5^ m^2^/s. From (12), a crystallization time t_cr_~10^−5^ s can be obtained. Considering that the velocity of the shock wave propagation is close to the speed of sound, we can estimate the time of particle spreading after the collision to be ~10^−7^–10^−8^ s. It can be concluded that the spreading out and crystallization of particles deposited on the substrate occur on a much shorter timescale than the incoming of the subsequent particles. Therefore, the ceramic synthesis via the gas-thermal synthesis process can be represented by the following scheme (Figure 7).

### 3.5. Radioluminescence Characteristics of β-Ga_2_O_3_ Ceramics

**Figure 8:**
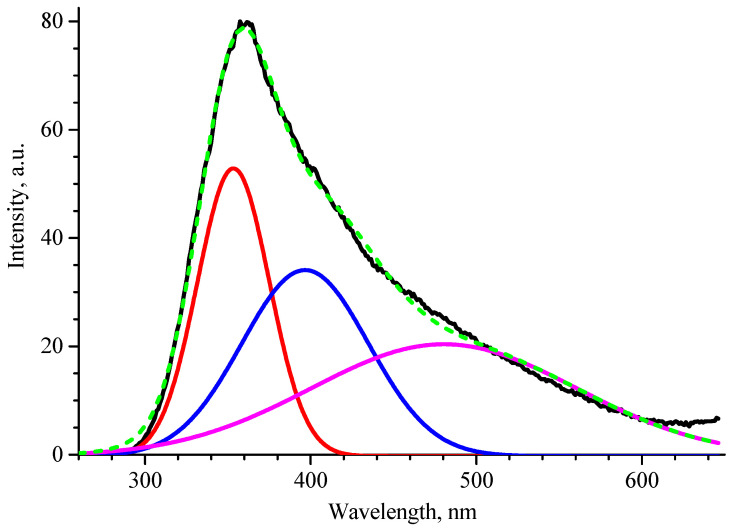
Radioluminescence spectra of β-Ga_2_O_3_ ceramics (black line) and its deconvolution (red, blue, magenta and dashed green as the cumulative curve).

Figure 8 shows the RL spectra of β-Ga_2_O_3_ ceramics. The maximum of intensity is located at ~360 nm wavelength. Its complex shape suggests the presence of several unresolved peaks. As a result of deconvolution, three bands were distinguished, with maxima at 353 nm, 396 nm and 480 nm. The highest-intensity band at 353 nm can be attributed to transitions between conduction band electrons and two differentially populated self-trapped hole (STH) states [30]. The band at 396 nm is considered to have appeared due to the recombination of free electrons and self-trapped holes [31]. The weakest band at 480 nm is most probably determined by transfer by tunnel effect of an electron from a donor cluster to a hole trapped at an acceptor site [32].

**Figure 9:**
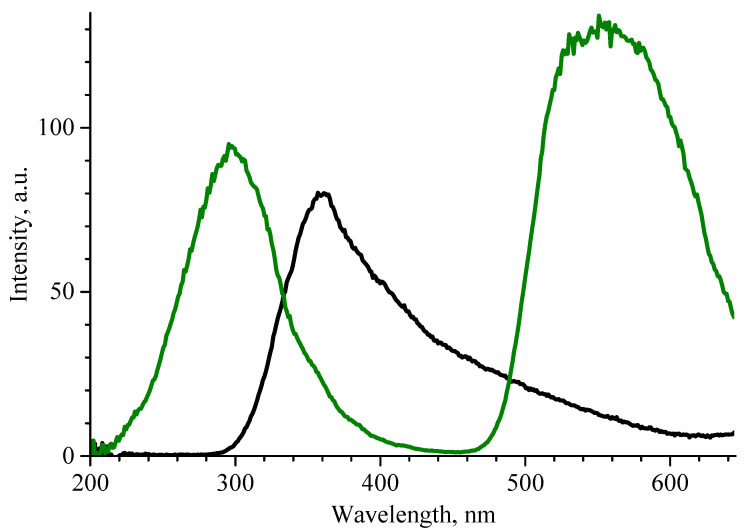
RL spectra of β-Ga_2_O_3_ ceramics (black line) and YAG:Ce crystal (dark green line).

Due to the complex shape, insufficient thickness, and low transparency of the β-Ga_2_O_3_ ceramic, direct measurement of its output was impossible. It is possible though to estimate the light yield using a sample with a known value. Figure 9 shows the RL spectrum of the β-Ga_2_O_3_ sample alongside that of previously studied spectra of YAG:Ce crystals [23]. Knowing that the total light yield of the studied YAG:Ce crystals was approximately 14,000 photons/MeV, one can calculate (based on the ratio of the areas under RL spectra) that the light output of the β-Ga_2_O_3_ ceramic is around 6100 photons/MeV. The given estimate is quite rough though, since it does not take into account either the difference in excitation intensity or the difference in the energy of the exciting quanta between the measurement of radioluminescence and light output.

**Figure 10:**
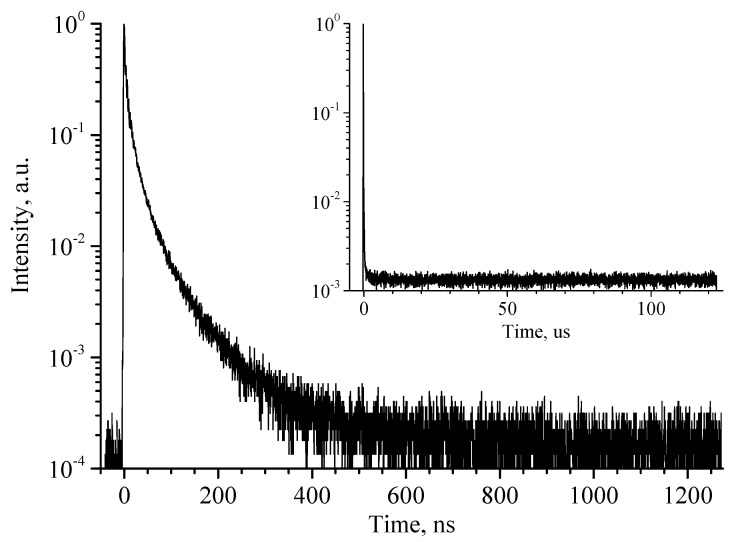
RL decay kinetics of β-Ga_2_O_3_ ceramics. Inset shows decay in a wider time frame.

The RL decay curves of β-Ga_2_O_3_ ceramics are shown in Figure 10. It consists of a relatively fast component of a non-exponential shape and a prolonged afterglow. The afterglow remains constant even in the widest available time frame (~125 us), indicating that its decay time is on the order of milliseconds. The fast component is non-exponential, covers ~600 ns from the start of the excitation pulse and makes up less than 30% of the integral signal.

## 4. Conclusions

This paper demonstrates the first results on the use of low-temperature plasma for the synthesis of ceramic materials. β-Ga_2_O_3_ ceramic samples were synthesized via the gas-thermal method utilizing a low-temperature plasma of an argon and nitrogen mixture. A remarkable synthesis rate of ~2 μm/s was achieved. A direct current electric arc generator of high-enthalpy plasma jet with a self-aligning arc length and an expanding channel of an output electrode served as a plasma source. The starting material consisted of a polydisperse powder of monocrystalline β-Ga_2_O_3_ with particle sizes ranging from 5 to 50 μm. The work presents the results of theoretical and experimental analysis of the velocity and average heating temperature of gallium oxide particles in a plasma jet depending on the gas mass flow rate. A model for the gas-thermal synthesis of β-Ga_2_O_3_ ceramics is proposed. The synthesized ceramic samples were characterized using scanning electron microscopy and X-ray diffraction methods. It was shown that during the process of synthesis, ceramics with a laminated microstructure are formed. The stoichiometric composition of the ceramics was found to be shifted towards the surplus of gallium. According to the data on X-ray diffraction, the values of lattice parameters and unit cell volume are decreased in β-Ga_2_O_3_ ceramic samples. The ceramic samples have an open porosity of approximately 5%. The RL spectrum of β-Ga_2_O_3_ ceramics features an intense emission band in the range from 300 to 600 nm, with the maximum at ~360 nm. The RL decay curve is non-exponential and has a relatively fast component (0–600 ns), contributing less than 30% of the total signal. The rest of the curve is a very long afterglow which, due to the limited time frame, cannot be characterized by a specific time constant. The light yield can be estimated to be around 6000 photons/MeV. In the future, the synthesized β-Ga_2_O_3_ ceramics may find application in scintillation detectors.

This study was carried out within the framework of a grant from the Russian Science Foundation (No. 21-79-30062 in terms of obtaining samples by the method of electric arc gas-thermal spraying synthesis), as well as within the state assignment of NRC “Kurchatov institute” and the Joint Institute for High Temperatures of the Russian Academy of Sciences (JIHT RAS No. 075-00270-24-00) in terms of theoretical and experimental studies of heterogeneous plasma and the obtained samples.

## Figures and Tables

**Table 1:** Composition of samples.

	Element	Atom, %
Initial powder	O	60.52
	Ga	39.48
Ceramics β-Ga_2_O_3_	O	55.71
	Ga	44.29

**Table 2:** Average size of CSRs of samples.

Direction	D of Initial Powder, nm	D of Ceramics β-Ga_2_O_3_, nm
[200]	34.6	62.0
[111]	24.4	60.0
[202]	22.5	53.6
[113]	22.6	51.8

**Table 3:** Crystallographic parameters of samples.

Lattice Parameters	Initial Powder	Ceramic β-Ga_2_O_3_	Ga_2_O_3_ [27]
a, A	12.145	12.177	12.214
b, A	5.774	5.794	5.798
c, A	3.034	3.033	3.037
β, degree	103.521	103.829	103.830
V, A^3^	2.068	2.078	2.088

## Data Availability

The original contributions presented in the study are included in the article, further inquiries can be directed to the corresponding authors.
